# Betwixt playing the waiting game and waiting in vain: Temporal governance and the thin alignment of care under universal health coverage in Kenya

**DOI:** 10.1111/maq.70074

**Published:** 2026-06-02

**Authors:** Edwin Ambani Ameso

**Affiliations:** ^1^ Faculty of Theology: Centre for African Studies University of Copenhagen København Denmark

**Keywords:** care infrastructure, Kenya, temporal governance, universal health coverage, waiting

## Abstract

This article investigates how Kenyan citizens access healthcare within the framework of Universal Health Coverage (UHC) reforms. Based on ethnographic fieldwork, it reconceptualizes waiting as a politically structured phenomenon rather than a simple delay. The analysis shows that UHC reforms do not eliminate waiting but instead redistribute it, resulting in new bureaucratic bottlenecks, infrastructural limitations, and unpredictable care timelines. The concept of ‘thin alignment’ is introduced to characterize a temporally unstable condition in which endurance and improvisational strategies coexist as patients navigate fragmented healthcare systems. Individuals depend on kinship networks, informal payments, religious practices, and social connections to make their suffering visible and actionable. By emphasizing temporality, this article illustrates how UHC reconfigures access to care and informs broader discussions on infrastructure, inequality, and the politics of care.

## INTRODUCTION

Universal Health Coverage (UHC) in Kenya aimed to remove financial barriers to care and to include marginalized citizens in the insurance system. However, for many, reform did not eliminate delay; it reorganized it. Based on 12 months of ethnographic fieldwork (2019–2020) in Kisumu and Kitui counties, I argue that UHC redistributed rather than resolved waiting, creating new bureaucratic bottlenecks, infrastructure breakdowns, and uncertain timelines for care. I introduce the concept of “thin alignment,” a temporally unstable condition in which waiting (endurance) and not waiting (improvisational action) co‐occur and shape each other. These practices do not arise in sequence but appear simultaneously within institutional constraints, requiring patients to endure delays and to try to interrupt them actively. In this context, waiting is more than temporal delay; it is a politically structured condition that reproduces subordination and recalibrates agency. By focusing on temporality, this article re‐examines UHC reform and adds to medical anthropological debates on infrastructure, inequality, and the politics of care.

In October 2019, when I met *Jaduong* Onyango^1^ he was accompanied by his able‐bodied son, David^2^, and his wife, Mama David, who patiently sat next to him. Jaduong Onyango was among the numerous patients occupying outpatient waiting spaces after an early Monday morning journey to the Kombewa County Referral Hospital (KCRH) in Kisumu County. Strategically located on the border of three counties in western Kenya, the facility offers health services to a wide population. Jaduong Onyango and his family had taken a 45‐min taxi ride to the hospital, beginning early in a bid to beat the long queues for much‐needed care. According to the hospital administration, Mondays were the busiest days of the week as patients and clients routinely attended their Tuberculosis (TB) and HIV/AIDS clinics, underwent triaging, and got medication refills. Other patients attended diabetic, maternal and child clinics.

With authorization, I observed patients and took the opportunity to (re)introduce myself to available health staff. The triaging health records officer drew my attention to the elderly Jaduong Onyango. Constantly shifting from side to side, stretching his body, and placing some of his weight onto his hand‐crafted wooden cane, Jaduong Onyango was discernibly in pain. Every motion seemed to cause suffering, a slight grimace on his face as he endured the wait for care. In his left hand, there was a bluish, eco‐friendly bag (in compliance with the state's Environmental Act, which bans plastic bags); a colourless tube snaked from the bag to Jaduong Onyango's lower right pelvic region. Jaduong Onyango, as I came to learn, was a prostatitis patient.

Jaduong Onyango's case offers insight into the waiting experiences that ordinary Kenyan citizens face when seeking health care. These individuals must navigate unstable public health settings, even as the state aspires to provide health as a public good through Universal Health Coverage (UHC). Prolonged waits for diagnosis or treatment often worsen health conditions and increase reliance on kin and other social support networks. Drawing from my interlocutors, I conceptualize waiting and not waiting as responses that are only loosely or superficially aligned to regimes of scarcity, uncertainty, and uneven access. Both are oriented toward care and survival, but they differ in practice and consequence. Waiting and not waiting are defining features for citizens experiencing political and economic subordination as they navigate UHC rhetoric within an unstable healthcare system.

Establishing productive linkages between technical know‐how and technical know‐who can transform waiting from failure to productivity in crisis‐laden healthcare contexts. This shift is evident as care seekers navigate institutional scenarios, relying on improvisation and social navigation as elements of care (Dobler, 2020). These dynamics are seen in the experiences of Jaduong Onyango and Mama N, an older woman in Kitui county whose prolonged wait for diagnosis and treatment ultimately led to the cessation of curative care. Together, these cases illustrate trajectories of waiting under UHC.

In Kenya's under‐resourced healthcare settings, individuals like Jaduong Onyango encounter failures and waiting as routine—missed appointments, stalled referrals, prolonged delays, and uncertain timelines. Patients and caregivers respond by mobilizing technical know‐how—understanding care procedures, paperwork, and clinical requirements. They also draw on technical know‐who—relationships with staff, intermediaries, and social networks—to make their health needs visible. These linkages are produced, not given, as patients and caregivers seek to prevent lost access and develop workarounds when systems falter. Through such innovativeness, waiting becomes a condition that enables the creation of alternative care pathways. Waiting reveals how care unfolds amid institutional protocols, queues, and inconsistent referral chains, fostering context‐specific social navigation. Living amid persistent breakdowns and uncertainties in the healthcare system requires continually recalibrating one's position within and against unstable biomedical systems structured by delays, scarcity, and workarounds. Waiting thus reveals the emotional experiences behind protracted failures in care access. It shows how fragile kinship care networks are mobilized to provide alternative access in the face of systemic failure.

Most scholarship on waiting has focused on asylum, displacement, or the temporalities of traditional livelihoods, where delays depend on state recognition, seasonal cycles, or economic survival (Hage, 2009; Andersson, 2014; Griffiths, 2014). However, its links to health reform remain underexplored. Through my Kenyan interlocutors, I move beyond previous focuses to examine waiting within healthcare systems and the future expectations of health. Here, waiting is not directed at legal outcomes or maintaining livelihoods, but at uncertainties in diagnoses, deferred treatment, care system breakdowns, workarounds, and hopes for recovery or fears of decline. Power dynamics in waiting are pervasive, disenfranchising poor citizens dependent on the state and generating feelings of powerlessness, helplessness, hopelessness, and vulnerability (Ameso, 2022; Lee et al., 2020; Sutton et al., 2011). My interlocutors often felt ‘less than human’ after prolonged waiting, their invisibility leaving them feeling undeserving of necessary care. Ultimately, they questioned their fundamental right to health as citizens deserving of equality and quality care amid ongoing breakdowns in the care infrastructure. Through their stories, I examine UHC reforms that reorganize rather than eliminate waiting. This produces thin alignment between waiting and not waiting, creating tactical improvisation and social navigation within fragile health systems (Livingston, 2012; Vigh, 2009). In this way, I critique UHC in Kenya, showing how it may worsen access and offering insight into how political subordination is reproduced (Auyero, 2012).

This article examines three interrelated dynamics shaping healthcare access under UHC: first, the temporal delays that structure access to diagnosis and treatment; second, the role of kinship networks and social support in navigating these delays; and third, the consequences of deferred care, including disease progression, financial strain, and moral recalibration. These dynamics are explored through ethnographic cases that demonstrate how patients navigate care within conditions of uncertainty.

While many studies focus on waiting in migration and bureaucratic contexts, fewer look at how space and infrastructure shape waiting in healthcare. In clinics, waiting happens in real places—lines, benches, and triage areas—where some are seen, and others are not. By examining how waiting occurs in these places, I show how time and space together shape healthcare access.

## THE PROMISE OF UHC REFORM AND TEMPORAL GOVERNANCE IN KENYA

From Dec 2018 to Dec 2019, the Kenyan government experimented with different interventions and health reforms to expand access to healthcare and financial protection for its citizens. In 2019, these national‐level reforms took the form of an experimental UHC rollout in four counties: Kisumu, Nyeri, Machakos, and Isiolo (Ameso & Prince 2022; Ameso, 2022). At the sub‐national level, some counties, enabled by the devolution of health service delivery to county governments and aligned with constitutional mandates and Kenya's Vision 2030^3^, experimented with independently led, county‐level health insurance initiatives, most notably in Kitui County. The national experiments were time‐limited, offering a year of care access across the experimenting counties, to scale up. In the four nationally chosen counties, free healthcare services were made available to residents, while in the independently experimenting county of Kitui, a county health insurance, Kitui County Health Insurance cover (K‐CHIC), was initiated with a 10 US dollar annual premium, covering health services as well as zero‐rating access to mortuary services for residents.

In the push to establish health sustainability in relation to sustainable development goals (SDGs), nation‐states in the Global South like Kenya, which have faced a high burden of diseases and impoverishing health expenditures for its citizens, began to turn to UHC as a key health priority and promise (Barasa et al., 2018; Ameso, 2022). With more than 40 per cent of total health expenditure in low‐ and middle‐income countries (LMICs) out‐of‐pocket, poor and vulnerable citizens often bear the highest burden of disease, leading to catastrophic expenditures (Fenny et al., 2021). In Africa, UHC's aspiration to ensure that all citizens receive the quality health services they need without suffering financial hardship reflects the bold political commitments embedded in the Africa Health Strategy 2016–2030 and the Addis Ababa call to action on UHC 2019, which African leaders have advocated (Cashin & Dossou, 2021).

Kenya's political commitment to achieve UHC by 2022 led to a focus on reforming the National Hospital Insurance Fund (NHIF)^4^, including the health insurance subsidy scheme for the poor (HISP) and other measures that might address the health expenditures that impoverish poor and vulnerable citizens (Barasa et al., 2018). Restructuring the NHIF offers fodder for political rhetoric advancing the utopian agenda of achieving UHC (Ndetei, 2021). In Kenya, the focus on health financing reforms has positioned the NHIF and social protection and welfare schemes like *Inua Jamii* cash transfers as central in the transformation of citizens' health needs^5^. Through cash transfer programs, such as those to the elderly, the promise of bringing poor and vulnerable populations into the health insurance fold offers relief to a population heavily impoverished by chronic illnesses and often left out of available biomedical gazes (Street, 2014; Ameso, 2022). However, the disbursement of these cash transfers through contracted commercial banks has often been delayed by lengthy processes and bureaucratic hurdles.

For years, Kenya has tinkered with various healthcare financing options to restructure the NHIF. These have been attempts to address allegations of corruption and organizational dysfunction that hinder the provision of health as a public good, and to transition to a national social health insurance (NSHI) that offers affordable, quality care to all citizens (Koon et al., 2020). However, achieving UHC requires strategically addressing not only healthcare financing but also health system service delivery. The unstable biomedical infrastructure available to ordinary citizens is prone to breakdown and underfunding and needs to be effectively equipped to address citizens’ needs (Street, 2014; Ameso, 2022). Jaduong Onyango and others’ narratives speak to living in a time oriented to and manipulated by institutional and bureaucratic actors—state agencies, insurance regimes, and healthcare administrators. These actors redistribute time and delay as techniques of health provision, forcing ordinary citizens to wait (Auyero, 2012). In the following vignettes, waiting emerges as an integral part of healthcare experiences in Kenya, characterizing overburdened public settings and leading care seekers to turn to options such as private care or ‘under‐the‐table’ payments and services to make their diseased bodies visible to necessary clinical gazes. In Kenya, waiting is an experience of social navigation in which human life is intricately woven with political choices, rhetoric, and aspirations to leapfrog decades‐long legacies of poor health (Göttlich 2015; Janeja & Bandak, 2018).

## FIELDWORK AND METHODS

My ethnography addresses reforms in the Kenyan public health system, specifically efforts to expand access (making healthcare reachable to more people) and coverage (broadening who is included) for all citizens (Ameso, 2022). I focus on health insurance (programs that help pay for medical expenses) and social protection (policies designed to reduce poverty and vulnerability) as key reforms and trace how their impacts unfold in diverse ‘waiting sites’—from health facility queues and cash transfer disbursement zones to state offices and homes. To explore Kenya's universal health coverage (UHC) experiments, which aim to provide health services to all without financial hardship, I conducted 12 months of multi‐sited ethnographic fieldwork from February 2019 to February 2020. My two primary sites, Kitui and Kisumu counties—representing the eastern and western regions—illuminated UHC experiments at both subnational and national levels (Ameso, 2022; Prince, 2022). Within these contexts, I concentrated on spaces where underinsured (those with insufficient insurance) and uninsured (those with no insurance) citizens wait for social services such as healthcare and (un)conditional cash transfers, meaning with or without requirements. I visited patients like Jaduong Onyango at home and during clinic visits, accompanying their families as they navigated the healthcare system.

The material presented follows health insurance and social protection objects as they are engaged by patients, families, social workers, and health workers within the government's efforts to expand care. Drawing on ethnographic interviews, informal conversations, and participant observation in health facilities and community settings, the article demonstrates how recruitment of interlocutors through clinic‐based encounters and community referrals reflects the relational and situated nature of care access. Fieldwork was mainly conducted in Kiswahili and local dialects, with translation provided as needed. My position as a young, early‐career Kenyan researcher shaped both my access and the narratives shared with me, particularly concerning experiences of waiting, authority, and care. Interlocutors positioned me as a ‘son,’ capable of making the realities of their navigation through the system visible to authorities. Attending to these dynamics was central to the unfolding of ethnographic encounters and to analyzing how UHC produced claims about care, time, and power.

From hospital waiting bays to the corridors of commercial banks and constituency offices where cash transfers were disbursed, my interlocutors and I oscillated between waiting and coming to terms with not waiting. Participant observation in clinical and community settings enabled close engagement with patients’ experiences of care seeking, including their negotiation and navigation of delays, institutional processes, and improvisations through social networks (Ameso, 2022). Waiting made visible the temporal and relational labor through which care was sought and delivered, revealing it as a transitory and transformative space that intersected with individuals’ life stages and social status, shaped by the material conditions of ongoing health reforms (Sutton et al., 2011). Waiting with patients, caregivers, and health workers revealed the subtle ways through which hopes, expectations, and hierarchies were perpetuated through social relationship formations, crystallizing critical liminal experiences to redress unmet health needs (Waltz, 2017). Waiting also allowed me to be actively co‐opted and engaged in social processes that reconfigured the everyday experiences of patients, health workers, hospital orderlies, and government functionaries. Through waiting with interlocutors, their experiences of waiting for healthcare became vivid; hopelessness, helplessness, and anger emerged as emotive entanglements for waiters, myself included. I accompanied the interlocutors in their experiences, anticipation, and despair as they tried to make sense of and rationalize the inaccessibility of care. To ground these conceptual claims, the remainder of the article turns to the two ethnographic cases of Jaduong Onyango and Mama N, illuminating how waiting and not waiting are thinly aligned in practice as interlocutors navigate care across fragmented healthcare infrastructures.

## JADUONG ONYANGO'S PROSTATITIS: REDISTRIBUTED WAITING UNDER UHC

After being diagnosed with prostatitis in March 2019 at Russia Hospital (a national teaching and referral hospital)^6^Jaduong Onyango began a prolonged and uncertain care trajectory. Complications necessitated multiple referrals and reconstructive surgery to address urinary tract damage. However, persistent delays, including postponed referrals, overbooked clinics, and extensive paperwork, repeatedly deferred access to care. Jaduong Onyango's experience highlights the central argument: under Universal Health Coverage (UHC), waiting is not merely incidental but is redistributed as a core feature of care, requiring patients to navigate and negotiate access within fragmented healthcare systems.

Although UHC promised access to reconstructive surgery, Jaduong Onyango and his family waited for care at the county referral hospital, which, like KCRH and others, lacked the capacity to provide the procedure. The family was compelled to wait strategically for an opening at Russia Hospital. While UHC reforms eliminated out‐of‐pocket costs for surgical procedures, they did not expand surgical capacity. Instead, increased demand intensified congestion at the Russia Hospital. The resulting long waitlist necessitated stopgap measures that redistributed, rather than eliminated, waiting. For several months, the family alternated between hope for surgery and reliance on routine bi‐weekly catheter replacements. After introducing myself, I joined the family for observations at home and during clinical visits. I waited in queues with them and made over‐the‐counter purchases, such as *Evac Lactulose* solution, for Jaduong Onyango. As the year progressed, shortages of medical supplies and breakdowns in care infrastructure forced the family to seek private health facilities to restore his health.

Jaduong Onyango became a frequent visitor to multiple public health facilities. Each visit entailed hours of queuing, triage delays, and time spent moving between departments while awaiting surgery. Concurrently, recurring health worker strikes, driven by overwhelming service demand and delayed pay, disrupted care nationwide. These strikes, along with stock‐outs and postponed surgical lists, repeatedly interrupted procedures, complicating Jaduong Onyango's access to care and rendering him largely invisible (Ameso, 2022). Throughout this period, the family monitored media reports about unpredictable health worker strikes. For instance, in August 2019, a scheduled doctors’ strike led to the cancellation of a long‐awaited operation, further prolonging uncertainty about reconstructive surgery.

During the 4 months after I first met him (October 2019–January 2020), I accompanied Jaduong Onyango and his family as they pursued elusive surgical care. This process involved frequent clinical visits to Russia Hospital and KCRH for biweekly catheter placements. To minimize the costs associated with private appointments and catheter replacements, the family increasingly relied on services at Russia Hospital under UHC coverage. Each catheter replacement visit required 5–6 h at the hospital, in addition to approximately 2 h of travel. Family members made five trips to the Russia Hospital, each lasting 7–8 h. Navigating these visits involved both formal referral pathways and informal coordination with hospital orderlies. Orderlies provided guidance on clinic schedules, facilitated movement between departments, and indicated when and where to seek clinicians' attention. Through these relationships, the family moved between regional and county referral hospitals, selectively reducing some waiting times while enduring others.

On a typical morning during these 4 months, the family and I arrived at Russia Hospital at dawn, joining other patients from western Kenya and beyond. The first stop was the triage zone, a small tent adjacent to the casualty and emergency unit entrance. We waited for the blood pressure machine, which had been loaned to trauma ward staff for monitoring emergency hemorrhage patients. During this time, Mama David, Jaduong Onyango's wife, went to the UHC registration area and returned with a small UHC receipt. After nearly 2 h, a blood pressure machine became available, and the triage nurse attended to Jaduong Onyango. Without triage sheets, the nurse recorded vital signs on the UHC receipt, enabling Jaduong Onyango to proceed to the doctor's consultation room. A brief examination resulted in a prescription, also written on the UHC receipt, directing the family to the pharmacy department. Seeking a catheter replacement, the family also attempted to secure another surgical appointment after a doctor's strike had prevented the initial one. Due to stock‐outs at the pharmacy, the family relied on what local bureaucrats described as “mushrooming” chemical shops: small, privately run pharmacies that have proliferated around public hospitals in response to frequent shortages. David and I visited these chemical stores and pharmacies to obtain the necessary drugs. With our early arrival, we also used the waiting time to address Jaduong Onyango's nutritional needs, often visiting the hospital restaurant for porridge. Meanwhile, Mama David waited at the surgical bay but was ultimately turned away, as health worker strikes led to further postponements (as later confirmed by hospital administration). The disappointment and frustration among those waiting was palpable; as Mama David expressed, “we are children of a lesser God,” left without care and made to wait in vain.

Waiting did not equate to passivity. To actively wait, David and I hurried to the outpatient wing to rebook surgery, perpetuating the cycle. Jaduong Onyango's extended search for surgery underscores a critical point: UHC reforms redistributed waiting times rather than eliminating them. New temporal bottlenecks emerged, where promised access coexisted with persistent scarcity and uncertainty. Families like his were compelled to endure, strategise, and improvise care over extended periods. His experience demonstrates that waiting is both imposed by the healthcare system and actively navigated by patients. Jaduong Onyango's case reveals the tenuous balance between waiting and not waiting. Waiting is enforced through strikes, stock‐outs, and referral bottlenecks, while not waiting is achieved through tactical navigation and informal coordination. These stopgap measures temporarily restore care but do not resolve underlying uncertainty. Jaduong's experience illustrates how UHC reconfigured the experience of waiting. Families must alternate between endurance and tactical improvisation to render suffering visible. UHC created a paradox: while zero‐rating reduced out‐of‐pocket costs, it intensified temporal bottlenecks within fragile care infrastructures. Jaduong Onyango's care depended on this delicate balance between waiting and not waiting, with institutional delays and tactical efforts closely intertwined. Waiting became a politically structured condition, necessitating constant recalibration. Families were required to endure uncertainty while improvising visibility within unstable healthcare systems.

## MAMA N'S INEVITABLE END: WHEN WAITING BECOMES FUTILE

In April 2019, when I met Mama N, an octogenarian widow, at her home in Kitui South sub‐county, an arid and semi‐arid rural setting, she was sitting on a small, worn‐out mattress. She looked frail, unable to sit up straight and relying on the adjacent house wall to maintain her posture. Swarms of flies surrounded the bluish *leso* scarf, carefully covering three‐quarters of her now‐malformed face and head^7^. The scarf helped fend off the flies, which circled her as the serosanguineous fluid seeped from the tip of her enlarged tumor, the size of a newborn baby's head. As her daughter‐in‐law and caregiver, Leah, informed me, the tumor had begun as a simple lump behind her earlobe in 2016.

Alex, a social worker with a non‐governmental organization, first brought Mama N's case to my attention during an informal conversation. He worked with vulnerable groups, particularly children from impoverished families, facilitating their access to education and healthcare through the organization's Angel project. Accompanying him on his daily visits to Angel program beneficiaries often led to discussions about additional cases of need shared by community members, such as Mama N's. Alex agreed to accompany me to visit her household.

Mama N was enrolled in the *Inua Jamii* older persons cash transfer program, which entitled her to a monthly stipend of 20 US dollars and National Hospital Insurance Fund (NHIF) coverage. When I met her, she had been waiting 7 months for her stipend to be disbursed. As she expressed, “I am waiting but hopelessly also not waiting.” The delay also rendered her NHIF coverage inactive, since premiums linked to the transfer had not been remitted. To cope, her family sold goats and cattle to pay for her care, but the proceeds were insufficient to cover her medical expenses. After a previous disbursement, Mama N used 10 US dollars (Ksh 1000) to pay the annual premium and subscribe to the county‐led health insurance, K‐CHIC. However, the geographical limitations of K‐CHIC and the lack of advanced diagnostic technologies at county health facilities meant that referrals stalled and her disease progressed without intervention.

Launched in late 2018, K‐CHIC sought to expand access to curative, preventive, promotive, rehabilitative, and selected specialized services across public health facilities, targeting more than 270,000 households. This county‐level initiative was supported by collaboration among government departments, aligning social assistance and health financing to enable the remittance of insurance premiums for cash transfer beneficiaries and to include poor and rural, underserved citizens in the insurance system (Barasa et al., 2018; Obare et al., 2014; Rabut, 2019). Through such mechanisms, K‐CHIC and similar county‐based insurance schemes aimed to address persistent access gaps in underserved areas and advance universal health coverage (UHC) through pro‐poor service delivery.

Despite K‐CHIC coverage, geographical and financial constraints meant that the cost of necessary diagnostics fell to relatives, particularly her son Peter, who worked in the *Juakali* 8 sector in Nairobi County. Attempts to access care produced only partial results. K‐CHIC provided access to the Kitui County Referral Hospital, but the inability to diagnose her condition required the family to seek alternatives. Although universal health coverage (UHC) services were zero‐rated in neighboring Machakos County and a K‐CHIC–covered ambulance facilitated her transfer, the absence of advanced diagnostic technologies meant the wait for care persisted. Medical practitioners recommended referral to Kenyatta National Hospital (KNH), where either an active NHIF status or out‐of‐pocket payments were necessary, both of which were unaffordable for the family. Consequently, the family relied on mobilizing relatives and selling livestock to raise funds for diagnostic tests at KNH.

As delays mounted, the family faced diminishing options. Leah, her daughter‐in‐law and caregiver, monitored radio announcements and contacted welfare offices to track cash transfer disbursement schedules. However, bureaucratic processes overshadowed clinical urgency. By the time the cash transfers arrived, Mama N had accepted her inevitable end. She allocated part of the funds to renew K‐CHIC, not for treatment, but to secure mortuary coverage. The prolonged wait underscored the irreversibility of Mama N's cancer. Erratic NHIF premium remittance, linked to delayed Inua Jamii disbursements, contributed to the progression of her suspected cancer, resulting in jaw dislocation and her left eye barely remaining in its socket. Leah suspected Mama N was in the terminal stage; a sense of hopelessness was evident as she continued caregiving. Leah and others had to accept the futility of their efforts, with the lack of accessible palliative and hospice care further sealing Mama N's fate.

When the *Inua Jamii* cash transfers finally arrived 7 months later, Mama N had become resigned to her fate. She chose to retain part of the 140 US dollars (Ksh 14,000) to protect her family from further impoverishment due to health expenses. While awaiting the inevitable, Mama N relied on over‐the‐counter medication to alleviate discomfort and pain. She paid the 10 US dollar annual subscription fee for K‐CHIC, which would later cover her embalming and a seven‐day zero‐rated stay at the only available sub‐county hospital mortuary. Mama N confronted her fate with resolve, expressing hope for the afterlife. As she told me, “I have lived a full life. [All I want you to do is] tell my story and that of others who suffer as they wait for health. For me, I am ready to go and meet my maker.”

My interactions with Mama N highlight the fragility of life and provide insight into how individuals navigate waiting, chronic illness, and health uncertainties. Persistent stock‐outs in public health facilities and the absence of advanced diagnostic technologies forced families to cope with uncertain realities, shifting waiting from hopeful anticipation to resignation (Ameso, 2022). The prolonged wait for care led to improvised palliative and hospice strategies, or what scholars have termed increased chronic homework—the extended, often unrecognized labor of caregiving at home (Mattingly et al., 2011). Despite her frailty, Mama N remained a source of support to her family, caring for her grandchildren as Leah took on the role of caregiver. Her resignation, as health uncertainties overwhelmed improvised care, represented a moral recalibration in the face of terminal delay rather than passive waiting. This acceptance reflected the recognition that pursuing curative care further would impoverish the household without improving outcomes. The subtle distinction between waiting and not waiting illustrates how temporal governance shapes both access to care and decisions about when to cease pursuing medical interventions.

## MODALITIES OF WAITING: INFRASTRUCTURES, KINSHIP, AND IMPROVISATION

Building on the ethnographic cases, this section identifies recurring modalities through which patients engage delays, institutions, and social networks to access care. Individuals waiting for healthcare, such as Mama N and Jaduong Onyango, engage with diverse temporal social formations and individualized patterns of waiting. They alternate between passive and active waiting as they navigate fragmented healthcare systems, often turning to private pharmacies to circumvent persistent stock‐outs in public facilities and queuing in hospital corridors for care. The aspiration to become visible to the biomedical gaze, despite its erratic and delayed care, underpins the waiter's capacity for improvisation (Ayaß, 2020; Janeja & Bandak, 2018). These experiences provide insight into the forms of action, thought, and social relationships that arise from various engagements with waiting and the temporal anxiety endured in unstable biomedical contexts (Debele, 2020; Street, 2014; Janeja & Bandak, 2018). Through routine interactions with public care infrastructures, modalities of waiting—temporal practices shaped by queues, referrals, documentation, and institutional recognition—become apparent as patients endure and negotiate bureaucratic processes to access care (Carswell et al., 2019).

Modalities of waiting further reveal how individuals strategically navigate care while enduring catastrophic health expenditures, despite the promises of Universal Health Coverage (UHC) (Ameso, 2022; Njagi et al., 2020). Waiting becomes a significant interstitial period, imbued with multiple meanings from the perspectives of ordinary citizens (Gasparini, 1995). These modalities expose the deficiencies of healthcare infrastructures as patients confront inadequate logistics in clinical settings. From improvising during clinical registration to coping with persistent stock‐outs, patients must determine how to situate themselves within institutional timelines and reconfigure their trajectories to access care. For example, in Jaduong Onyango's case, waiting for care at the Russia hospital involved addressing additional co‐morbidities; his family and I engaged the laboratory department to obtain a malaria diagnosis while simultaneously awaiting his elusive surgery.

For my interlocutors, waiting constituted more than a productive process; it became “a state of consciousness, where one is unceasingly thinking about the wait while also constantly updating oneself of the social and political conditions waiting has imposed” (Ambujam, 2023, 1). While waiting alongside them, I observed that Mama N and Jaduong Onyango became deeply entangled with bureaucratic processes and unstable biomedical attention in their efforts to address their health needs (Abadia & Oviedo, 2009). Interlocutors consistently sought to remain informed about available care alternatives through their social networks. Through these social formations of waiting, individuals became informed health seekers, closely monitoring local media coverage to navigate health worker strikes and stock‐outs that intensified access barriers and rendered healthcare unattainable (Ayaß, 2020; Ameso & Prince, 2022).

Enduring long queues at health facilities and *Inua Jamii* cash transfer collection points and offices further illustrate the power dynamics inherent in waiting, as well as how connections formed during these periods of uncertainty can facilitate networks of care. While accompanying Mama N and Jaduong Onyango, I observed them cycle through emotions such as anger, disappointment, powerlessness, vulnerability, and hopelessness, as the persistent invisibility of their illnesses complicated their access to care. From waiting for social cash transfers and insurance premium remittances to confronting the compounded realities of being under‐ or uninsured, interlocutors navigate the power structures that shape their experiences of waiting (Ameso, 2022). Waiting reinforces institutional hierarchies and redefines the positionality of healthcare seekers: some achieve temporary visibility through tactical engagement, others remain marginalized, and all must negotiate, improvise, and assert agency within bureaucratic care processes.

Waiting for, and subsequently becoming invisible to, healthcare underscores how neoliberal ideologies of affordable and quality care for all—implemented by incorporating ordinary and vulnerable citizens into insurance schemes—reinforce interlocutors' marginalization, positioning them among the poor (Fogarty & Cronin, 2008; Lee et al., 2020). As members of this group, they depended on access to care in order to accept the imposed roles of waiting, submission, and adaptation to the dominance of health reforms. Although health insurance and social protection reforms are intended to transform the healthcare landscape and care‐seeking trajectories (Janeja & Bandak, 2018), my interlocutors' experiences reveal that these reforms often exacerbate existing gaps. Bureaucratic interventions associated with UHC reforms delayed or prevented care, resulting in catastrophic outcomes (Lee et al., 2020). Mama N was significantly affected by these bureaucratic processes and timelines, which determined her limited access to care and forced her to confront the constraints of waiting. Bureaucratic delays in disbursing cash transfers and remitting health insurance premiums prevented Mama N from optimizing her healthcare‐seeking strategies.

Within these ethnographic accounts, waiting emerges as a more‐than‐human condition, intricately linked with hospitals, medical devices, bureaucratic procedures, and social assistance programs that shape the temporal rhythms of care. In this context, waiting functions as both a concept and a metaphor: conceptually, it illustrates how fragile infrastructures and institutional hierarchies mediate patients’ bodies, agency, and visibility; metaphorically, it signifies the prolonged, uncertain, and complex pathways through which ordinary citizens navigate health and livelihood in precarious environments. Furthermore, the inability of social cash transfers to address the health needs of indigent care seekers, as Mama N highlights, underscores the power structures and politics of health reforms that disenfranchise vulnerable populations (Auyero, 2012). As Mama N and Jaduong Onyango experienced anticipation, doubt, hopefulness, and a liminal wait for the inevitable, they ultimately became resigned to the cessation of waiting.

## WAITING'S ENTWINEMENTS: IMPROVISATION AND SOCIAL SUPPORT

My encounters with Jaduong Onyango highlighted how waiting in clinical settings is shaped by systemic dysfunction, mistrust, and insufficient application of technologies designed to manage patient flow. Clinical visits were often interrupted by health worker strikes and technological breakdowns, including malfunctioning triage, X‐ray, and CT equipment, as well as a reliance on paper records when digital systems failed. These systemic and technological shortcomings substantially increased waiting times for health services. Extended waiting periods resulted in adverse outcomes for interlocutors, including exacerbation of co‐morbidities and reduced quality of life. Efforts to improvise solutions to these delays frequently exhausted families' already limited resources (Brown and Creswell, 2006), reinforcing the argument that deficiencies in healthcare delivery directly contribute to negative patient outcomes and resource depletion.

Epidemiological transitions intersect with persistent waiting and familial vulnerabilities, exposing the inadequacy of standard care timelines within Kenya's unstable biomedical infrastructure. In these contexts, improvisation and social navigation become essential strategies for accessing care. The central argument is that the anticipated benefits of medical modernization and state‐led development frequently remain unrealized, compelling citizens to address unmet health needs within complex healthcare systems (Ameso, 2022; Stasik et al., 2020; Vigh, 2006). Active waiting not only highlights the marginalized status of these patients but also imposes significant burdens on their families, who must assume caregiving responsibilities in the absence of reliable services (Mattingly et al., 2011). For instance, Mama N's reliance on kin networks for palliative care demonstrates how families mobilize social resources to compensate for insufficient clinical attention (Street, 2014).

The significant financial burdens experienced by individuals such as Mama N illustrate persistent inequalities in healthcare access (Ilinca et al., 2019). During periods of waiting, individuals like Jaduong Onyango may resort to informal payments to expedite care. Jaduong Onyango attempted to arrange biweekly catheter replacements and urgent surgery by informally coordinating with hospital orderlies. Families sometimes provided small, unrecorded payments or gifts to accelerate care and attract attention, thereby circumventing bureaucratic obstacles. For those with life‐threatening conditions, such as Mama N, waiting is a constant reality. Disease progression and escalating health expenses often conflict with other household needs, increasing the risk of impoverishment. For both Mama N and Jaduong Onyango, waiting became a distinct temporal experience, shaped by their efforts to navigate social, political, and personal challenges in accessing care. Mama N benefited from clan welfare, which provided support to all elderly members without requiring fees. Jaduong Onyango's son, David, enlisted the assistance of his sister, Millicent, a teacher. As kin‐based support became insufficient, families established broader solidarity groups on social media platforms (primarily WhatsApp^9^) to mobilize additional resources. Thus, active waiting is a pervasive feature of chronic illness, as families organize care through kinship networks (Lee et al., 2020).

During periods of waiting, Mama N and Jaduong Onyango developed social connections that influenced their evolving identities. Individuals with chronic illnesses, shaped by broader political shortcomings, endured slow and limited care as their conditions deteriorated. For instance, Mama N received care from her daughter‐in‐law, while Jaduong Onyango relied on close relatives. These relationships transformed their daily experiences and contributed to the formation of new identities (Debele, 2020). During my home visits, extended family members frequently gathered to discuss strategies for managing waiting periods. Mama N's caregiver, Leah, monitored fund disbursement schedules, maintained contact with the social welfare office, and tracked relevant radio announcements. She also investigated the extent of coverage provided by county health insurance (K‐CHIC) after exhausting the most recent social cash transfer. Jaduong Onyango's family navigated public health systems that were insufficiently equipped to address such cases. As the health status of Mama N and Jaduong Onyango remained challenging for clinicians to assess, health workers' improvisational efforts also influenced the experience of waiting. Consequently, waiting for care emerged as a dual process involving both care seekers and providers, both of whom were entangled in systemic delays (Hage, 2009).

As state‐provided healthcare becomes increasingly unreliable, families assume a central role in care provision, positioning informal kin networks as the foundation of health support systems (Gleeson et al., 2016). The central argument is that proximity to familial and social networks is crucial for navigating care, yet limited resources and shifting responsibilities strain these relationships. In Mama N's family, financial constraints and competing household demands required caregivers to balance improvised care with other obligations. The growing burden on caregivers and delays in cash transfers highlight the vulnerability of family‐based care amid systemic failures and unmet needs.

Waiting periods became opportunities for individuals to construct new identities. Many coped with healthcare challenges by engaging in religious practices and prayer. Family members also placed hope in these practices while awaiting care (Debele, 2020). For example, during one visit, Jaduong Onyango's sister travelled from her home; although unable to provide financial assistance, she prayed for his endurance during the waiting period. Similarly, Mama N's relatives prayed as they faced delays and high healthcare costs. Religion and prayer offered frameworks for understanding their experiences and fostered social and financial support networks. These practices facilitated the development of alternative caregiving strategies. Additionally, informal payments occasionally attracted clinical attention to those in need (Ameso, 2022).

The distinction between waiting and not waiting delineates time into distinct periods, revealing the emergence of bureaucratic challenges such as stock‐outs, routine delays, and postponed care. These issues also exemplify what some bureaucrats refer to as the politics of tenderpreneurs^10^ where discrepancies between expected and actual health procurement practices become apparent (Stasik et al., 2020), for families, waiting and not waiting represent more than the passage of time; they integrate lived experience with analytical significance. These processes expose the structural violence embedded in healthcare systems. Waiting further illuminates the ongoing labor of caregiving, described as “chronic homework,” and highlights the limitations of family support (Gandorfer & Ayub, 2021; Mattingly et al., 2011).

## CONCLUSION

Waiting constitutes a pervasive reality in both clinical and non‐clinical contexts. Chronically ill and economically disadvantaged patients encounter health workers’ improvisations, uncertainty, and powerlessness, which shape their daily lives. Extended waiting becomes inevitable, influencing routines and shaping expectations for future health. The temporalities of waiting are closely intertwined with health imaginaries, as prolonged delays and unpredictable schedules recalibrate expectations for care and well‐being. This dynamic highlights tensions between lived experiences and anticipated outcomes. Within this context, the concept of thin alignment—the oscillation between waiting and not waiting—emerges as a critical analytical lens. Patients and citizens must continually negotiate when to wait and when to act. Waiting is both imposed and strategically navigated. Enduring delays and tactical improvisation demonstrate how agency can emerge within structural constraints. Waiting is imposed through strikes, stock‐outs, referral bottlenecks, and delayed cash transfers, while not waiting results from informal coordination, kin mobilization, shadow payments, and, at times, moral resignation. Poor and vulnerable citizens embed these strategies in their daily navigation of care. These actions reveal the politics of waiting and the creative labor required to access services in Kenya's resource‐constrained settings. Rather than being oppositional, waiting and not waiting represent interdependent strategies for navigating care.

Centering temporality reframes the evaluation of Universal Health Coverage (UHC). Policy assessments typically emphasize enrolment, financial protection, or service coverage, yet rarely address temporal access—the capacity to obtain timely diagnosis, treatment, and recognition. In unstable health systems, reforms may increase insurance membership while simultaneously deepening temporal inequalities. These experiences underscore the persistent gap between the promises of UHC and the realities patients face, which are often characterized by uncertainty, despair, and mistrust. Chronic waiting reflects broader structural constraints and exposes the state's limited capacity to provide timely care. On a global scale, neoliberal reforms have shifted states from providers of public goods to service facilitators, fundamentally shaping the health landscape that citizens must navigate. In Kenya, the vision of UHC remains largely aspirational and utopian, as waiting lists frequently restrict access to even basic services. Thus, waiting both reveals and recalibrates the practical limits of universalism.

Following the 2019–2020 fieldwork, reductions in international funding for Kenyan and African health initiatives have intensified pressure on public health infrastructure. These developments have likely exacerbated chronic waiting, deepened inequities in access to care, and increased reliance on improvisational strategies. Such pressures further strain the thin alignment between waiting and not waiting. By centering the experiences of ordinary and vulnerable health seekers, this analysis demonstrates that waiting is temporal, conceptual, and relational. It reflects structural inequalities and shapes the strategies employed to navigate them. Understanding thin alignment is essential for evaluating both the promises and limitations of UHC in Kenya, particularly in the context of global austerity and limited public resources.

For medical anthropologists and scholars, this analysis underscores how health infrastructure is experienced temporally. Bureaucratic delays, technological failures, and fiscal austerity generate lived uncertainty, shaping subject formation and shifting care responsibilities onto households. Temporality is not merely an adjunct to access; it fundamentally shapes it. As global health actors confront fiscal pressures and renewed austerity, attention to the structural production of waiting becomes increasingly urgent. The promise of universality risks devolving into empty rhetoric if temporal bottlenecks persist. Examining how citizens navigate the boundary between waiting and not waiting reveals UHC as both a financing reform and a temporal regime. This dynamic determines whose suffering and visibility are deferred, and whose lives are transformed by waiting.

## CONFLICT OF INTEREST STATEMENT

The author declares none.

## ENDNOTES

1 Jaduong is a term from the Dholuo community that refers to an older man.

2 David and other names used in this article are pseudonyms to adhere to ethical standards of anonymity and confidentiality.

3 In Kenya, ‘devolution’ refers to the transfer of responsibility for health service delivery from national to county governments following the promulgation of the 2010 constitution, which was implemented in 2013. While the national government retains oversight of health policy and regulation, counties manage public health facilities, health personnel, and local health‐financing initiatives.

4 NHIF is the oldest health insurance in sub‐Saharan Africa, and Kenyan citizens utilise it to access care (see Barasa et al., 2018).

5
*Inua Jamii* is the government's flagship National Safety Net program under the Ministry of Labour and Social Protection and the Ministry of Devolution and ASAL, covering beneficiaries of orphans and vulnerable children (CT‐OVC), older persons cash transfer (OPCT), persons with severe disabilities (PWSD‐CT), and the Hunger Safety Net programme (HSNP) (see Mwasiaji 2015; UON WEE Hub, 2022).

6 Russia Hospital is a level 6 hospital designed and built in postcolonial Kenya by Soviet architects, planners, and aid as part of a friendship cultivated by then‐opposition political figure Oginga Odinga in Kisumu County (see Prince 2020).

7 Leso refers to a printed fabric common in eastern Africa and often worn by women.

8 Juakali refers to the informal sector of small traders and businesspeople who operate in the open markets and streets of urban areas.

9 WhatsApp is a social media app that lets people create online groups for exclusive communication.

10 Tenderpreneur is a portmanteau of “tender,” to provide business services, and “entrepreneur.” It refers to a businessperson who uses political contacts to secure government procurement contracts, or tenders, often as part of a reciprocal exchange of favours or benefits (see Piper and Charman 2018).
